# Recent advances in understanding and managing cholesterol gallstones

**DOI:** 10.12688/f1000research.15505.1

**Published:** 2018-09-24

**Authors:** Agostino Di Ciaula, Piero Portincasa

**Affiliations:** 1 Division of Internal Medicine - Hospital of Bisceglie, ASL BAT, Bisceglie, Italy; 2 Clinica Medica “A. Murri”, Department of Biomedical Sciences & Human Oncology, University of Bari Medical School, Bari, Italy

**Keywords:** cholesterol gallstones, gallbladder, bile, pathogenesis, primary prevention

## Abstract

The high prevalence of cholesterol gallstones, the availability of new information about pathogenesis, and the relevant health costs due to the management of cholelithiasis in both children and adults contribute to a growing interest in this disease. From an epidemiologic point of view, the risk of gallstones has been associated with higher risk of incident ischemic heart disease, total mortality, and disease-specific mortality (including cancer) independently from the presence of traditional risk factors such as body weight, lifestyle, diabetes, and dyslipidemia. This evidence points to the existence of complex pathogenic pathways linking the occurrence of gallstones to altered systemic homeostasis involving multiple organs and dynamics. In fact, the formation of gallstones is secondary to local factors strictly dependent on the gallbladder (that is, impaired smooth muscle function, wall inflammation, and intraluminal mucin accumulation) and bile (that is, supersaturation in cholesterol and precipitation of solid crystals) but also to “extra-gallbladder” features such as gene polymorphism, epigenetic factors, expression and activity of nuclear receptors, hormonal factors (in particular, insulin resistance), multi-level alterations in cholesterol metabolism, altered intestinal motility, and variations in gut microbiota. Of note, the majority of these factors are potentially manageable. Thus, cholelithiasis appears as the expression of systemic unbalances that, besides the classic therapeutic approaches to patients with clinical evidence of symptomatic disease or complications (surgery and, in a small subgroup of subjects, oral litholysis with bile acids), could be managed with tools oriented to primary prevention (changes in diet and lifestyle and pharmacologic prevention in subgroups at high risk), and there could be relevant implications in reducing both prevalence and health costs.

## Introduction

Gallstone disease is highly predominant in Western countries, where it has a prevalence of up to 15% in adults ^1^ and is one of the most common causes of hospital admission for gastrointestinal disease in European countries ^2^. Also, gallstone disease has high health costs, especially in the presence of gallstones that become symptomatic or cause complications ^1, 3, 4^. The presence of gallstones also generates concerns when occurring in children ^5^ and adolescents (<20 years of age) ^6^. Though less frequent than in adults, the prevalence of cholelithiasis in childhood is increasing. In England, an increased incidence of cholecystectomy (from 0.78 in 1997 to 2.7 per 100,000 in 2012) has been reported in children not more than 16 years old ^7^. In a Canadian population-based, retrospective cohort, the crude incidence of cholecystectomy in subjects under 18 years of age increased from 8.8 per 100,000 person-years in 1993 to 13.0 per 100,000 person-years in 2012 ^8^, and a retrospective study in the US over a nine-year period ending in 2012 registered an increment of cholecystectomies due to pediatric non-hemolytic (cholesterol) gallstones of 216% ^9^. It has been suggested that this epidemiologic increment during childhood could be due to the increasing prevalence of obesity ^6, 10– 16^, physical inactivity, diabetes, and early pregnancy ^6^.

Cholesterol gallstones are found in more than 80% of patients with gallstone, and the pathogenesis involves both local (that is, gallbladder and bile) and systemic factors ^17^.

Elevated levels of pro-inflammatory proteins such as interleukin-6 (IL-6), IL-10, IL-12(p70), and IL-13 appear to be associated with the risk of gallstones ^18^. Data from epidemiologic studies suggest an association between gallstone disease and higher risk of incident ischemic heart disease ^19, 20^ and total mortality and disease-specific mortality (cardiovascular disease and cancer). These links were statistically confirmed after adjustment for potential confounders, including traditional risk factors (such as body weight, cigarette smoking, physical activity, diabetes, hypertension, and hypercholesterolemia) ^19– 22^. Thus, complex systemic pathways might link the development of gallstones to other metabolic abnormalities (that is, insulin resistance, obesity, type 2 diabetes, non-alcoholic fatty liver disease, and metabolic syndrome itself) ^23– 32^. The association might also involve extra-gallbladder tumors ^33, 34^ in the liver ^35^, stomach ^36^, and colon ^37^. In this scenario, cholesterol gallstone disease becomes the expression of systemic metabolic and non-metabolic abnormalities ^38^.

## Pathogenic clues for adequate management of gallstone disease

Critical factors contributing to the formation of cholesterol gallstones are defective gallbladder motility, hypersecretion and accumulation of mucin gel in the gallbladder lumen with ongoing local immune-mediated inflammation, rapid phase transition of cholesterol from supersaturated hepatic bile, and precipitation of solid cholesterol crystals ^17, 39^. Additional features include gene polymorphism, increased hepatic cholesterol secretion, increased absorption of biliary and dietary cholesterol, sluggish intestinal motility, and qualitative, quantitative, or topographic changes of gut microbiota ^17, 40^. The complex and variable interactions of such pathogenic factors contributing to cholesterol cholelithiasis require a comprehensive discussion to correctly address the management of the disease.

The relevance of a genetic background in the pathogenesis of cholesterol gallstones is disclosed by studies addressing the family history of cholelithiasis ^41, 42^, evaluating selected ethnic groups ^43, 44^, and confirming the presence of specific gene polymorphisms ^45– 61^. The simple existence of predisposing genetic conditions, however, is not sufficient to promote cholesterol gallstone formation. This concept is supported by studies on twin pairs, where genetic factors play a role in no more than 25 to 30% of subjects with symptomatic gallstones ^62, 63^. Indeed, genes provide an increased risk of forming gallstones, but several environmental factors such as diet and physical activity ^58, 64– 66^ but also exposure to chemicals as heavy metals ^67, 68^ or pesticides ^69, 70^ (which also involve epigenetic mechanisms ^17, 71^) must play a crucial additional role in determining how many subjects will effectively develop gallstones. This aspect is particularly relevant for primary prevention by acting on environmental factors.

**Figure 1.**
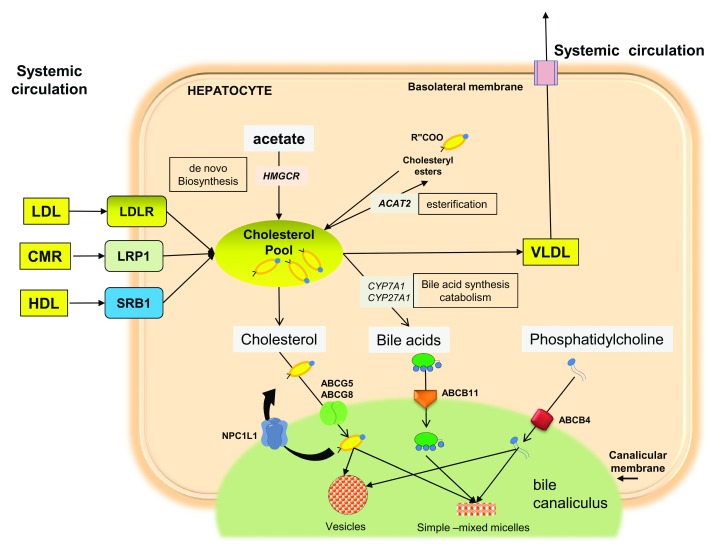
Pathways of cholesterol metabolism in the hepatocyte in humans. Cholesterol can enter the hepatocytes as low-density lipoprotein (LDL) via the LDL receptor (LDLR), as chylomicron remnants (CMRs) via the prolow-density lipoprotein receptor-related protein 1 (LRP1), and as high-density lipoprotein (HDL) via the scavenger receptor class B member 1 (SRB1). The absorbed cholesterol and the cholesterol synthetized from acetate via the rate-limiting enzyme 3 hydroxy 3 methylglutaryl-coenzyme A reductase (HMGCR) contribute to the intrahepatic cholesterol pool. From here, cholesterol can follow different pathways: esterification (by the acyl-coenzyme A: cholesterol acyltransferase isoform 2, ACAT or SOAT1) and storage in the hepatocyte; incorporation into assembled very-low-density lipoprotein (VLDL) and secretion; catabolic synthesis of bile acids—via the rate-limiting enzymes cholesterol 7α hydroxylase (CYP7A1) and sterol 27 hydroxylase (CYP27A1)—and secretion in bile; and direct secretion into bile. Lipid secretion in bile requires distinct ATP-binding cassette (ABC) transporters at the canalicular membrane of the hepatocytes: the heterodimer ABCG5/G8 for cholesterol, ABCB11 for bile acids, and ABCB4 for phosphatidylcholine. In humans, NPC1L1 is at the canalicular membrane of hepatocytes and is responsible for the reuptake of cholesterol from bile back into the hepatocytes. The nuclear receptors in the hepatocyte (not shown) play a key role at different levels: the farnesoid X receptor (FXR or NR1H1) is controlled via the intestinal fibroblast growth factor 19 (FGF19) and its receptor (FGFR4) in the liver and regulates bile acid synthesis through the small heterodimer partner (SHP) ^92, 93^. The liver X receptor (LXR or NR1H3) regulates cholesterol synthesis (via the cytochrome P450 51A1, or CYP51A1), bile acid synthesis (via the UDP glucuronosyltransferase 1–3, or UGT1A3), and biliary cholesterol secretion (via transcriptional activation of *ABCG5/G8*) ^94– 96^. One to three lipids (that is, cholesterol, bile acids, and phospholipids) are secreted into the bile canaliculus, and they aggregate into the typical cholesterol carriers—that is, simple (1–2 nm), mixed micelles (4–8 nm), and small unilamellar (40–100 nm) or large multilamellar (300–500 nm) vesicles ^97^.

Figure 1 depicts pathways of cholesterol metabolism in the hepatocyte in humans. Cholesterol gallstones grow in the gallbladder and are made of aggregated solid cholesterol crystals originating from bile supersaturated with cholesterol, which is no longer solubilized by micelles and vesicles ^40^. During the process of cholesterol cholelithiasis, the homeostasis of cholesterol is highly disrupted, and events involve cholesterol intake, metabolism, and synthesis. Defects involve the cholesterol transporters ABCG5 and ABCG8 (which pump sterols out of hepatocytes into the biliary ducts and from enterocytes into the intestinal lumen ^72^), hormones (that is, estrogen ^73, 74^, insulin ^30^, and thyroid hormones ^75^), nuclear receptors ^23, 76– 78^, and factors governing the entero-hepatic recirculation of cholesterol ^79– 81^.

Women display an increased prevalence of gallstones compared with men because of the influence of estrogen on cholesterol metabolism ^73^. This effect involves the enhanced synthesis of cholesterol and decreased synthesis of bile acids (BAs), which are sterols synthesized from cholesterol, and represents the main catabolic pathway of cholesterol metabolism in humans. The step involves the upregulation of estrogen receptor 1 and G protein–coupled receptor 30 ^82, 83^. A recent meta-analysis also confirmed a positive association between the intake of exogenous estrogen and the risk of cholelithiasis ^74^.

The pathogenesis of cholesterol gallstones is closely linked with frequent metabolic abnormalities involving insulin resistance ^30^, obesity, dyslipidemia (hypertrigliceridemia), type 2 diabetes ^44, 84^, and metabolic syndrome per se ^85– 90^. Insulin resistance produces several lithogenic effects; that is, it increases the activity of the rate-limiting step in cholesterol synthesis, hydroxyl-methyl-glutaryl coenzyme A reductase ^91^; modulates the expression of the *ABCG5* and *ABCG8* genes involved in the expression of cholesterol transporters (governing biliary cholesterol secretion); and dysregulates the liver transcription factor forkhead box protein O1 (FOXO1) ^94^, which modulates cholesterol homeostasis and high-density lipoprotein (HDL)-mediated reverse cholesterol transport to the liver ^98^. In the liver, insulin resistance influences the level and activity of the BA sensor nuclear receptor farnesoid X receptor (FXR) ^23, 76, 77^, which is involved in crucial pathways of cholesterol and BA metabolism. FXR also upregulates the hepatic expression of the *ABCG5* and *ABCG8* genes by activating the other BA sensor, liver X receptor (LXR) ^78^. Thus, FXR plays a protective role against the development of cholesterol gallstones and pharmacologic agents as 6-α-ethyl-ursodeoxycholic acid (6EUDCA) modulating the activity of this nuclear receptor could be a useful therapeutic tool ^99^. Also, results from animal models suggest that piperine (a potential cholesterol-lowering agent) could be useful in preventing cholesterol gallstone formation by inhibiting the expression of the cholesterol transporters ABCG5/8 and LXR ^100^.

**Figure 2.**
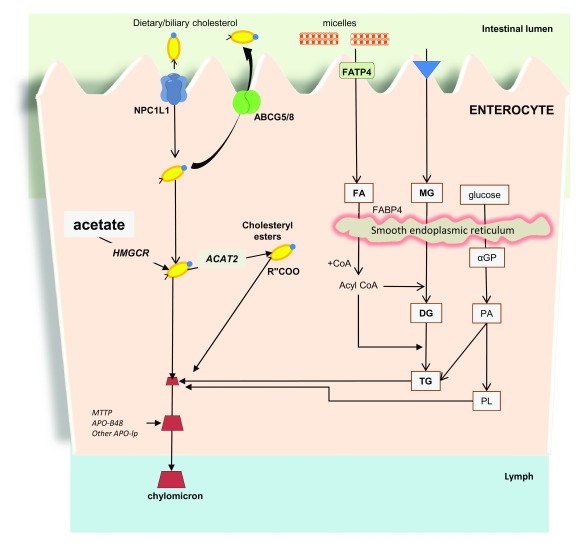
Regulation of intestinal cholesterol absorption and role of the Niemann-Pick C1-like 1 protein (NPC1L1) at the enterocyte level in humans. Bile acids enrich both hepatic and gallbladder bile. Sterols undergo micellar solubilization in the intestinal lumen, and uptake occurs at the enterocyte brush border. The NPC1L1, located at the apical membrane of the enterocytes, governs the active uptake of cholesterol and plant sterols across the brush border membrane of the enterocytes. Ezetimibe specifically inhibits the NPC1L1 pathway. The ATP-binding cassette transporters ABCG5/G8, also located at the enterocyte brush border, promote the active efflux of cholesterol and plant sterols from the enterocytes into the intestinal lumen for fecal excretion. In the enterocyte, new synthesis of cholesterol occurs from acetate (by 3-hydroxy-3-methylglutaryl-CoA reductase, or HMGCR). The esterification of intracellular cholesterol requires the acyl-CoA:cholesterol acyltransferase isoform 2 (ACAT2). Chylomicrons are assembled following triacylglycerol (TG) and phospholipid (PL) synthesis, steps requiring facilitated transport of fatty acids (FAs) and monoacylglycerol (MG) into the enterocytes, which include their FA binding protein 4 (FABP4)-mediated transport into the smooth endoplasmic reticulum. This step is followed by synthesis of diacylglycerol (DG) and TG, whereas PL synthesis is dependent on glucose transport into the smooth endoplasmic reticulum and synthesis of α-glycerophosphate (α-GP) and phosphatidic acid (PA). Apolipoprotein (APO)-B48 and activity of microsomal triglyceride transfer protein (MTTP) are required for assembly of chylomicrons before their secretion into the lymph. Thus, final chylomicron particles contain a core of triglycerides and cholesteryl esters surrounded by a surface enriched with PLs (mainly phosphatidylcholine), unesterified cholesterol, and apolipoproteins, including A-I, A-II, A-IV, B-48, C-I, and C-III. APO C-II and APO-E are acquired when the chylomicrons enter the circulation ^96, 97, 104^. Adapted from 105.

The systemic homeostasis of cholesterol is influenced by the small intestine; here, dietary cholesterol is absorbed ( Figure 2), and biliary cholesterol is reabsorbed during the entero-hepatic circulation of BA ^101^, with variable efficiency levels ^102, 103^. The step is co-regulated by genes determining the balance between influx of intraluminal cholesterol molecules crossing the enterocyte brush border membrane—via the Niemann-Pick C1-like 1 (NPC1L1) pathway—and efflux of enterocyte cholesterol into the intestinal lumen (via the ABCG5/ABCG8 pathway) ^101^. A large observational study showed that genetic variations in *ABCG5/8* associated with decreased levels of plasma low-density lipoprotein (LDL) cholesterol are protective against myocardial infarction but increase the risk of symptomatic gallstone disease, suggesting that there is an intrinsic link between these two diseases and that this link is based on the activity of ABCG5/8 ^106^. The coding variant rs11887534 (D19H) in *ABCG8* is associated with a more efficient transport of cholesterol into bile ^45^. Of note, despite the presence of D19H polymorphism ^107, 108^, patients with gallstones have significantly lower cholesterol absorption ^107, 108^ and higher ** or unchanged ^108^ *de novo* synthesis of cholesterol ^107^. This peculiar metabolic feature could precede gallstone formation in groups at risk ^107^. Independently from the presence of obesity, cholesterol absorption by the small intestine can be reduced by the presence of insulin resistance, which is also able to increase cholesterol synthesis ^109, 110^.

In animal studies, cholesterol gallstone formation can be prevented by ezetimibe, the selective inhibitor of the intestinal NPC1L1. The mechanism involves reduced amounts of absorbed cholesterol reaching the liver through the entero-hepatic circulation and, in turn, decreased biliary cholesterol saturation ^79– 81^. Additionally, deficiency of osteopontin (OPN) (a soluble cytokine and a matrix-associated protein detectable in the majority of tissues and body fluids) in OPN ^−/−^ mice on lithogenic diet reduces the intestinal absorption of cholesterol through a suppressed expression of NPC1L1, preventing cholesterol gallstone formation ^111^.

The potential effects of the intestinal microbiota on the pathogenesis of cholesterol gallstones should not be neglected. Gut bacterial communities collected from patients with gallstones exhibit increased phylum *Proteobacteria* and a decrement of genera *Faecalibacterium*, *Lachnospira*, and *Roseburia* ^112^. The cecum of patients with gallstones contains increased amounts of Gram-positive anaerobic bacteria with elevated 7α-dehydroxylation activity, a finding linked with increased concentrations of the more hydrophobic and lithogenic secondary BA deoxycholate ^113^. Increased concentrations of secondary BA could also depend on enrichment with the genus *Oscillospira*, negatively correlated with primary BA formation. An opposite trend exists for the phylum *Bacteroidetes* ^114^. A reduced richness and alpha diversity of microbiota, with lower levels of *Firmicutes* and decreased ratio of the phyla *Firmicutes* to *Bacteroidetes*, have been reported in mice fed a lithogenic diet and forming gallstones ^115^.

A number of studies have examined the role of defective gallbladder motility (that is, increased fasting and postprandial gallbladder volumes and slower postprandial emptying in response to meal) as a major risk factor for cholesterol gallstone formation ^44, 116– 119^. Indeed, intraluminal gallbladder stasis provides a sufficiently prolonged time for nucleation of excess biliary cholesterol and gallstone growth ^120– 122^. A sluggish motility function of the gallbladder also predisposes to gallstone recurrence after successful extracorporeal shock-wave lithotripsy or oral BA litholysis or both ^123, 124^. Abnormal gallbladder motor function is found in about one third of patients, independently of the physical presence of (small) stones ^125– 130^, and is a feature before gallstone occurrence ^120, 125, 131, 132^. Alterations in the gallbladder contractility are secondary, at least in part, to a direct toxic effect of unesterified biliary cholesterol on the gallbladder smooth muscle plasma membranes ^133– 135^. The steps involve the migration of intraluminal cholesterol into the muscularis propria, inhibition of action potentials, currents of Ca ^2+^ ^136^, decreased density of cholecystokinin-1R (CCK-1R) on the smooth muscle ^137^ and signal-transduction decoupling of the CCK-1R ^122, 138– 140^ (mainly due to CCK binding to cell receptors not followed by G-protein activation ^138, 141– 143^), and smooth muscle cell proliferation and inflammation in the gallbladder mucosa and lamina propria ^119, 144^.

A defective intrinsic innervation of the gallbladder has also been described with marked reduction of neurons, enteric glial cells, mast cells, and interstitial cells of Cajal in patients with gallstones ^145^. These alterations lead not only to altered postprandial contraction but also to defective interprandial relaxation ^146, 147^.

Of note, extra-gallbladder (that is, systemic) factors play a relevant role also in the modulation of fasting and postprandial gallbladder motility. Besides the existence of polymorphisms in the *CCK-1R* gene that might be associated with gallstone formation in humans ^148^, insulin resistance has been linked with defective gallbladder motility in lean, non-diabetic, and gallstone-free subjects ^149^. The gallbladder dysmotility is also described in women with polycystic ovary syndrome, a condition often linked with insulin resistance ^150^. Notably, the anti-diabetic agent metformin ameliorates the gallbladder motility defect ^151^, and prolonged therapy with this drug has been linked with reduced risk of gallstones in patients with diabetes ^152^.

The interprandial gallbladder relaxation leading to organ refilling is stimulated by the acid-induced duodenal release of vasointestinal peptide (VIP) and is regulated by the human protein fibroblast grow factor 19 (FGF19, FGF15 in mice) ^153^, located in the gallbladder epithelium but also in cholangiocytes ^154^ and in the ileum ^155^. Secreted BAs reach the terminal ileum and act as signaling factors, which activate FXR and, in turn, increase the intestinal FGF19 levels in the portal circulation. FGF19 binds to its receptor fibroblast growth factor receptor 4 (FGFR4)/co-receptor β-klotho found in the liver and also in the gallbladder ^156^. FGF19-FGFR4/β-klotho interaction in the gallbladder accounts for relaxation of the gallbladder smooth muscle and this feedback modulates gallbladder refilling between meals ^119, 153^. Intraluminal hydrophobic BAs also act as signaling agents for the G protein – coupled bile acid receptor 1 (GPBAR-1) ^157^, located in the gallbladder epithelium and smooth muscle ^156, 158^ and stimulating gallbladder relaxation independently of FGF19 ^159^, through the activation of KATP channels ^160^.

The existence of impaired gallbladder refilling due to altered interprandial motility might have a role in the pathogenesis of gallstones. In the intedigestive period, fasting rhythmic fluctuations physiologically decrease the gallbladder volume by 20 to 30% ^161, 162^. An altered interprandial gallbladder motility ^120, 163^, mainly secondary to less frequent migrating myoelectric complex cycles and abnormal motilin release, has been described in patients with cholesterol gallstone as compared with healthy subjects ^119, 125, 128, 163^. The interprandial motility defect would be able to increase the hepatic secretion of lithogenic bile to the small intestine, with faster recycling of BAs and increased hydrophobicity of the BA pool ^164^, a mechanism predisposing to cholesterol crystallization and stone growth ^165^. As suggested by animal studies, CCK incretion might also have a role in the modulation of fasting gallbladder motility linked with an impaired small intestinal motility since an increment of fasting gallbladder volume, a prolonged intestinal transit time, and an increased intestinal cholesterol absorption have been detected in CCK knockout mice ^166^.

## Future perspectives: from therapy to prevention

The majority of patients with gallstone disease remain asymptomatic throughout their life ^167, 168^ and should be managed with watchful waiting ^40^. If symptoms or complications occur or if subjects are at high clinical risk of complications, the current therapeutic approach is surgery, which remains the mainstay treatment ^169^, by laparoscopy, small incision, or (in selected cases) laparotomy ^40, 170^. In a small (10%) subgroup of patients with small (<5 mm), pure-cholesterol radiotransparent stones in a functioning gallbladder with a patent cystic duct, litholysis with oral ursodeoxycholic acid (UDCA) (12 to 15 mg/kg per day up to 12 to 24 months) could be considered ^40^. Nevertheless, the risk of post-dissolution gallstone recurrence is high (about 10% per year and up to 50% by five years ^171^).

Cholecystectomy has a low risk of mortality (30-day mortality 0.15%, close to that of the general population in a Swedish study ^172^) but is not a neutral event. About 10% of patients can develop non-specific gastrointestinal symptoms following cholecystectomy (that is, the “post-cholecystectomy syndrome”) mainly due, in the first three years, to gastric diseases, including peptic ulcer, hiatus hernia, and gastro-esophageal reflux. In these cases, however, a misdiagnosis of pre-existing clinical conditions is possible. In a longer time window, the most common cause is the presence of stones within the biliary tree ^173^.

Although laparoscopic cholecystectomy has a low surgical risk and is the most common elective abdominal surgery performed in the US ^174^, a series of complications is possible, ranging from conversion to open cholecystectomy (the most frequent) to bile leak, bile duct injury, and (in few cases) death ^175^. It was recently suggested that surgical complications are more likely in patients with low socioeconomic status, who appear to be more vulnerable. In fact, patients from low-income populations show higher rates of 30-day mortality, in-hospital complications, readmission for complications, hospital costs, and length of stay in comparison with the general population ^176^.

Increased health costs have also been described in the case of delayed cholecystectomy, as compared with early cholecystectomy ^177^, and of emergency surgical procedure ^178^ and laparoscopic cholecystectomy, as compared with small-incision open cholecystectomy (with similar quality of life after these last two procedures) ^179^.

The robotic-assisted laparoscopic cholecystectomy appears to be an interesting and emerging technology. However, in the case of benign gallbladder diseases, this procedure does not seem to be more effective or safer than conventional laparoscopic cholecystectomy, which should be preferred because of lower costs ^180^.

Finally, the potential metabolic consequences of cholecystectomy have recently been discussed. Cholecystectomy might disrupt trans-intestinal flow of BAs acting as signaling (hormonal) agents via FXR, GPBAR-1 intestinal receptors in the absence of their natural reservoir (the gallbladder) ^181^. This aspect has practical implications, and cholecystectomy is suggested after adequate selection of patients on the basis of clinical and epidemiological evidence. It is mandatory that the surgical procedure be restricted to patients with specific symptoms (that is, colicky pain) or complications of cholelithiasis or to subjects at high risk requiring prophylactic cholecystectomy ^181^.

In conclusion, given the limits, risks, and costs of the currently available therapeutic approaches, the critical role played by systemic factors in the risk of gallstone formation and in the pathogenesis of cholesterol gallstone disease might offer interesting possibilities for primary prevention ^64, 71, 105, 182, 183^ aimed at reducing gallstone prevalence in subjects at risk and the related health costs.

A major intervention in the general population should include lifestyle changes ^64, 184– 189^, including dietary models able to possibly reduce the risk of gallstones mainly acting on lipid metabolism and metabolic pathways leading to gallstone formation ^188^ (that is, a low-carbohydrate diet with enriched vegetable proteins ^188^, nuts ^190^, and vegetable oils ^191, 192^ with moderate alcohol intake ^193^), and adequate physical activity ^187^ aimed at maintaining a normal body weight.

In fact, the risk of developing gallstones appears to increase with some dietary factors (that is, increased energy intake, low dietary fiber content, highly refined sugars, high fructose and fat intake, and low vitamin C intake) and to decrease with others (that is, olive oil consumption, ω-3 fatty acids, high intake of monounsaturated fats and fiber, vegetables, vitamin C supplementation, fruit, and moderate alcohol consumption) ^64^. In a large French cohort, the high adherence to the Mediterranean diet was linked with a significantly lower risk of cholecystectomy ^185^. Physical activity also positively influences several metabolic features related with the hepatobiliary–gut axis, and beneficial effects are anticipated by regular physical activity on several metabolic disorders, including cholesterol gallstone disease ^184^.

**Figure 3.**
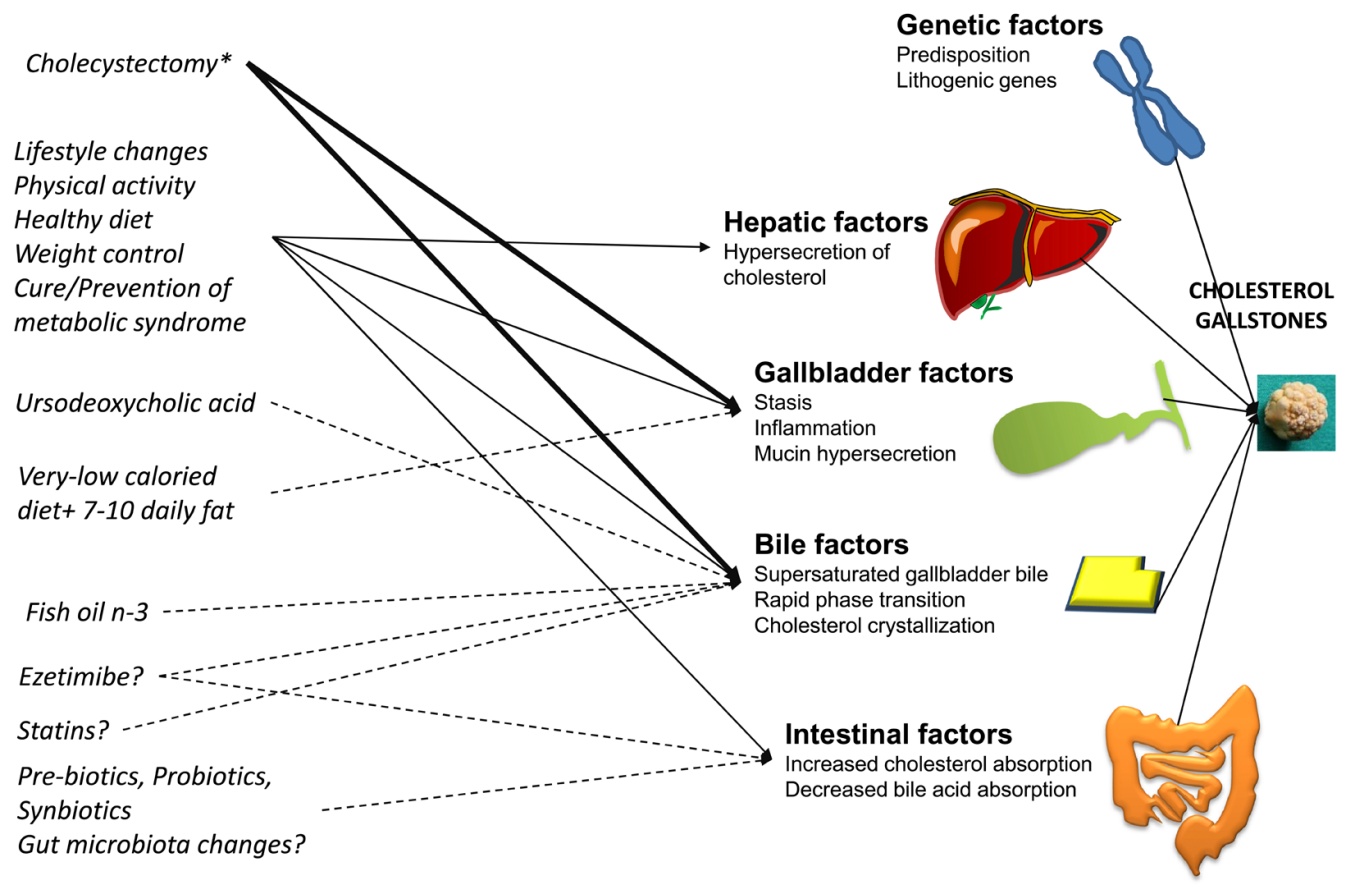
Pathogenetic factors involved in the formation of cholesterol gallstones and therapeutic opportunities. Increased hepatic hypersecretion of cholesterol represents the primary cause of cholesterol gallstone formation developing in the background of genetic predisposition. Gallbladder factors include defective motility due to a form of leiomyopathy developing in response to excess biliary cholesterol, local immune-mediated inflammation, and hypersecretion of mucin in the gallbladder lumen. Bile factors include the accumulation of supersaturated, concentrated gallbladder bile, a predisposing factor to rapid phase transitions of cholesterol to solid crystals, aggregation, and conglomeration within the mucin gel. Intestinal factors increase absorption of cholesterol and reduce absorption of bile acids. Therapeutic interventions include cholecystectomy, which radically cures gallbladder and bile abnormalities (and is effective in the case of pigment gallstones); general lifestyle recommendations; dietary changes; regular physical activity; and cure and prevention of metabolic abnormalities. Ursodeoxycholic acid is reserved to a subgroup of symptomatic uncomplicated gallstone patients with small stones, radiolucent (x-ray) in a functioning gallbladder and patent cystic duct. Also, in the high-risk group of patients undergoing rapid weight loss (that is, bariatric surgery or very-low calorie diet), ursodeoxycholic acid and a daily fat content of 7 to 10 g are recommended to improve gallbladder emptying and to prevent the formation of cholesterol gallstones following rapid weight reduction. All other therapeutic options are currently not supported by strong scientific evidence or require further studies ^40, 94, 105, 169, 200^.

Owing to the scarcity of strong evidence of effectiveness, pharmacological prevention of gallstones is not advisable in the general population ^169^. In theory, however, each pathogenic factor involved in gallstone formation could be considered a potential therapeutic target for prevention or treatment of cholesterol cholelithiasis ( Figure 3). In particular, a protective role is possible for the hydrophilic bile acid UDCA ^194– 199^. Other therapeutic options (alone or in combination) are promising but not yet supported by strong evidence: statins or inhibitors (or both) of the intestinal absorption of cholesterol such as ezetimibe ^79, 95, 201– 207^; nuclear receptor modulators ^99, 208– 212^ such as 6EUDCA, a synthetic derivative of UDCA acting as FXR modulator ^99^, or piperine, an alkaloid able to inhibit ABCG5/8 and LXR expression in the liver reducing biliary cholesterol secretion ^100^; possible modulators of gut microbiota ^112^; dietary/lifestyle models ^64, 65, 184, 213^; pharmacologic agents acting on gallbladder hypomotility ^121, 214, 215^; and inflammatory cytokine modulators ^216– 218^.

Future experimental and clinical studies are certainly needed to clarify the real usefulness of these potentially innovative preventive tools in selected groups of subjects.
